# Effects of parity, season of birth, and sex on within-litter variation and pre-weaning performance of F1 Large White × Landrace pigs

**DOI:** 10.14202/vetworld.2024.1459-1468

**Published:** 2024-07-07

**Authors:** Nqobile Lungile Buthelezi, Bohani Mtileni, Khathutshelo Agree Nephawe, Mamokoma Catherine Modiba, Hezekiel Mpedi, Peter Ayodeji Idowu, Takalani Judas Mpofu

**Affiliations:** 1 Department of Animal Sciences, Faculty of Science, Tshwane University of Technology, Private bag X680, Pretoria, 0001, South Africa; 2 Topigs Norsvin Animal Genetic Center, Farm Bossemanskraal 538 JR, Bronkhorstspruit, 1020, South Africa

**Keywords:** birth weight coefficient of variation, born alive, pig production, pre-weaning mortality, survival rate

## Abstract

**Background and Aim:**

A piglet’s pre-weaning performance significantly influences both animal welfare and profitability in pig production. Understanding piglet pre-weaning performance influencing factors is key to enhancing animal welfare, reducing losses, and boosting profitability. The study aimed to evaluate the impact of parity, season of birth, and sex on within-litter variation and pre-weaning performance of F1 Large White × Landrace pigs.

**Materials and Methods:**

Information regarding total litter size, number of born alive, number of stillbirths, piglet weight at birth, mortality, and count of weaned F1 Large White × Landrace piglets was acquired from the farm database (April 2022–February 2023). 2602 females and 2882 males, a total of 5484 piglets were utilized, with records from 360 sows. The coefficient of variation (CV) of birth weights among piglets within a litter was calculated. The general linear model analysis in MiniTab 17 was used to evaluate the data, with Fisher’s least significant difference test (p < 0.05) used for mean separation and Pearson’s moment correlation coefficient calculated to assess relationships between survival rates, mortality rates, litter size, birth weight, and birth weight CV.

**Results:**

Parity had a statistically significant impact on litter size, birth weight, and survival rate (p < 0.05). The sow’s parity did not significantly (p > 0.05) impact the number of piglets born alive or weaned. Multiparous sows had a significantly larger litter size (p < 0.05) than primiparous sows at birth. The litter weights for parities 2, 4, and 5 did not significantly differ (p > 0.05), with averages of 20.95, 20.74, and 20.03 kg, respectively. About 91.29% was the highest survival rate recorded in parity 2 (p < 0.05). The 1^st^ week of life recorded an 8.02% mortality rate. The mortality rate in parity 3–5 group was significantly (p < 0.05) higher (11.90%) in week 1 than in the other groups (parity 1: 6.79%, parity 2: 5.74%, parity 3–5: 8.54 and 9.21%). The litter sizes in autumn (17.34) and spring (17.72) were significantly larger (p < 0.05) than those in summer (16.47) and winter (16.83). In autumn and spring, the survival rate (83.15 and 85.84%, respectively) was significantly lower (p < 0.05) compared to summer (88.40%) and winter (89.07%). In all seasons, the litter weights did not significantly differ (p > 0.05). The birth weight CV was significantly (p < 0.05) lower during summer (20.11%) than during spring (22.43%), autumn (23.71%), and winter (21.69%). The season of birth had no significant effect (p > 0.05) on the number of live piglets. Males (1.34 kg) were heavier (p < 0.05) than females (1.30 kg) at birth. Notably, the birth weight CV was similar between males (22.43%) and females (22.52%). Litter size was positively correlated with average litter weight (rp = 0.576, p < 0.001), birth weight CV (rp = 0.244, p < 0.001), and mortality rate (rp = 0.378, p < 0.001). An insignificant relationship was observed between average litter weight and birth weight CV (rp = –0.028, p > 0.05) and survival rate (rp = –0.032, p > 0.05).

**Conclusion:**

In F1 Large White × Landrace pigs, birth uniformity among piglets declines as litter size grows larger. In parity 3–5, multiparous sows yield litters with reduced uniformity. With an increase in litter size, uniformity among piglets at birth worsens. A larger litter size and greater piglet birth weight variation are linked to a higher pre-weaning mortality rate. Producers need a balanced selection approach to boost litter size and must cull aging sows carefully to introduce younger, more productive females.

## Introduction

Pig production is one of the most important animal production industries in South Africa, and it plays a role in ensuring food security in the country [1]. The South African pig sector encompasses commercial and small-scale farmers, primarily producing pork for consumption. The industry is also involved in other pig-derived products, such as bacon, ham, and sausages, which are sold both domestically and internationally [2]. Approximately 2000 commercial pig producers operate in South Africa, as stated by the South African Pork Producers Organization. Pig numbers are estimated at 1.357 million for 2020, which is a decrease of 2.3% compared to 2019 [3]. A decrease in pork meat consumption may cause this. Lack of vaccination, biosecurity measures, and treatment of sick pigs might be responsible for the decrease in pig production [4]. High feed costs, diseases, and mortalities are among the hurdles confronted by this industry. [5]. Pig farming is a significant source of employment, especially in rural areas [6]. To meet competing demands, governments and industries must balance increased efficiency and productivity with food security, climate change adaptation, and high environmental and animal welfare standards [7]. Investigating causes of loss in the industry is crucial for enhancing the success of pig production in the country. The pre-weaning performance of pigs is one hurdle to overcome.

Understanding how factors such as parity, litter size, season of birth, and sex impact piglet pre-weaning performance is crucial for enhancing animal welfare, reducing losses, and maximizing profits in pig farming. The piglet pre-weaning performance is associated with the number of parities and variation in birth weight within the litter, which differs among parity [8–10]. On the other hand, the season of birth has been observed to be a problem in South Africa, negatively affecting not only the reproductive performance but also the economic efficiency of pig herds [11]. Temperature variations and photoperiodic reactions during seasons are considered the main drivers of fertility [12]. Seasonal infertility can be patent in several ways, but most reports have focused on reductions in the farrowing rate and litter size [13, 14]. Therefore, there is a need for selection programs that may prevent such issues (reduced farrowing rate) in their selection indexes. Commercial selection programs for sow performance have focused more on genetic improvements in litter size at birth [15], which have led to more live piglets [16]. Large litters produce high levels of birth weight variation, leading to poor survival due to high competition within litters for functional and productive teats [17, 18]. However, litter size remains an important trait in the pig industry because it determines the success of pig producers [19]. Large litters require more nutrients both before and after birth. Therefore, producers are encouraged to focus more on maintaining larger litters to minimize variations in birth weight within litters. Notably, the management of lower birth weight piglets, which have become more common with an increase in litter size, remains challenging [20]. Therefore, larger piglets may be more competitive than smaller piglets throughout the suckling period [21, 22]. Lower birth weight piglets tend to have a higher mortality risk, grow slower, and consequently need extended days to reach slaughter weight than their heavier littermates [23]. Pig producers manage this issue by offering lighter-weight piglets a special feeding regime [23, 24], cross-fostering [25], split suckling [26], and weaning them at a later age than their littermates [23]. Re-homing piglets can also be used to ensure litter uniformity [27] to facilitate management over the entire production period [16]. Birth weight and relative birth weight within a litter are the most essential variables that have an impact on mortality [28]. Pre-weaning mortality of piglets indicates both animal welfare [29] and economic problems [18] in the pig industry.

The effects of parity, season of birth, and sex on within-litter variation and pre-weaning performance in the F1 Large White × Landrace in South Africa are yet to be determined. The relationship between litter size, birth weight, and birth weight variation within litters, and survival and mortality rates, remains unclear. The objective of this investigation was to evaluate the impact of parity, season of birth, and sex on within-litter variation and early growth of F1 Large White × Landrace pig.

## Materials and Methods

### Ethical approval

The experimental procedures and animal handling were approved by the Tshwane University of Technology Animal Research Ethics Committee (AREC2023060020). This study was conducted in accordance with the ARRIVE guidelines (Animal Research: Reporting of *in vivo* Experiments) and the South African National Standards: (i) The South African Pig Welfare Code and (ii) the Care and Use of Animals for Scientific Purpose (SANS 10386:2008).

### Study period and location

The study was conducted from April 2022 to February 2023 at Topigs Norsvin, South Africa, which is situated in the Kungwini Local Municipality in the Gauteng Province. The farm is at a latitude of 25 48’ 36” South and a longitude of 28 44’ 32” East in a subtropical highland climate with dry winters. The location’s annual temperature is 22.1°C with a minimum temperature of 13.7°C and a maximum temperature of 26.2°C and has 100.9 rainy days (27.6%).

### Experimental animals and management

Data were extracted from the farm database (April 2022–February 2023) consisting of total piglets born, number of born alive, number of stillbirths, piglet weight at birth, mortality, and number of piglets weaned from the F1 Large White × Landrace. A total of 5,484 piglets (female: 2,602 and male: 2,882) from 360 sow litter records were used. The health of the animals was monitored daily, and illness was treated by trained personnel.

After weaning (day 21), all sows were moved to the service line and flushed. Sows in the dry sow crates (service) were flushed twice a day (morning and afternoon), on levels from 5 to 6 kg *ad libitum*. A teaser boar was placed in front of the sows to facilitate contact during the weaning-to-service period. When signs of heat were observed, sows were served with two post-cervical artificial insemination tubes 24 h apart. Briefly, the ejaculates used for artificial insemination were assessed using a computer-assisted semen analysis for morphology (>70%), motile per live cells (>65%), and progressive motility (>60%). Boar checks for returns were carried daily (returns stand for a boar ±21 days after service), and sows that returned were inseminated again. After 8 weeks in crates, sows were transferred to dry sow groups (9 sows per group) according to parity and backfat thickness (P2). During gestation, sows were fed the gestational diet (Table-1).

**Table-1:** Gestation diet of the sows.

Nutrients	Gestation diet	

	1–11 weeks of gestation	12–15 weeks of gestation
Crude protein (%)	15.5	16.4
ME (Kcal/kg)	3.048	3.048
Lysine (%)	0.9	0.9

ME=Metabolizable energy

Sows were placed in dry sow houses for 0–110 days of gestation and then moved to farrowing houses a week before the expected farrowing date. The sows were individually housed in fully slatted farrowing cages. Each farrowing crate had a single feeder with *ad libitum* access to water through a water nipple and a heat lamp for piglets. The farrowing houses were mechanically ventilated, and the temperature was maintained at approximately 18°C–20°C.

During the farrowing process, sows were monitored to categorize piglets into three groups: born alive, stillborn, and mummified. A piglet was considered alive if it displayed immediate movement and breathing after birth. Stillbirths were noted as fully developed piglets without any movement or heartbeat, while mummified fetuses were recorded but were not included in the analysis. The time between piglet births was also recorded to identify cases in which sowing assistance might be needed (birth intervals longer than 30–45 min). Once the sow finished farrowing, piglets were ear tagged with a unique tag number on the right ear, individually weighed on a 30-kg scale, and tail docked using a gas tail cutter approximately 2 cm from the base of the tail in between the 2^nd^ and a 3^rd^ vertebra. All sows received a lactation diet containing 18.1% crude protein (CP), 3.314 Kcal/kg metabolizable energy (ME), and 1.2% lysine. During lactation, sows were fed *ad libitum* until weaning. Mortality was recorded until weaning (21 days). In large litters, piglets were split and suckled, giving smaller, or late piglets a chance to consume adequate colostrum and were fostered within 12–24 h after birth to equalize the number of piglets with the number of functional teats. Within 3 days of age (DOA), piglets received 1 mL of iron with 1 mL of toltraboost (oral supplements) produced by Charles Street Veterinary Consultancy (South Africa) for deworming. Seven days later, all the piglets were tattooed on the ear. After 10 days, piglets were introduced to solid feed (creep feeding) to weaning. After weaning, the sows were returned to their gestational homes.

The following parameters were calculated:


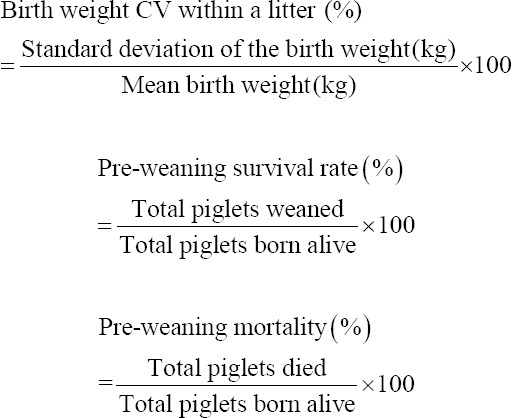


### Statistical analysis

Data were analyzed using a general linear model (GLM) in MiniTab 17 Statistical Software (MinTab Inc., PA, USA), and mean separation was carried out using Fisher’s least significant difference (LSD) test (p < 0.05). In addition, the Pearson Moment correlation coefficient was calculated to assess the relationship between survival and mortality rates with litter size, birth weight, and birth weight coefficient of variation (CV).

The following statistical model was used:


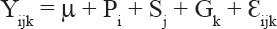


Where Y_ijk_ = Measurement of response (litter size, total born alive, number of born dead, birth weight, average litter weight at birth, birth weight CV, number of piglets weaned, mortality, and survival rate), μ = Overall mean, P_i_ = Fixed effect of parity (1, 2, 3, 4, 5), S_j_ = Fixed effect of season of birth (spring, summer, autumn, and winter), G_k_ = Fixed effect of sex of piglets, and ɛ_ijk_ = Random error.

## Results

The total litter size born ranged from 5 to 27 with an average of 16.60 piglets, and the piglets born alive ranged from 5 to 24 with an average of 15.24 piglets (Table-2). Much variation, as determined by the SD, existed in pre-weaning performance; the largest SD was for survival rate (10.27%), whereas the smallest SD was for birth weight (0.35 kg). The average birth weight at birth and birth weight CV were 1.34 kg and 21.24%, respectively. The numbers of weaned piglets ranged from 7 to 15, with an SD of 2.88 piglets. The number of piglets born dead ranged from 0 to 9, with a mean of 1.37 and SD of 1.61 piglets, respectively.

**Table-2:** Means, SD, Min and Max values for pre-weaning production performance of F1 Large White × Landrace piglets.

Variables	Number	Mean	SE mean	SD	Min	Max
Litter size	360	16.60	0.19	3.56	5.00	27.00
Piglets born alive	360	15.24	0.18	3.32	5.00	24.00
Born dead	360	1.37	0.09	1.61	0.00	9.00
Birth weight (kg)	5484	1.34	0.01	0.35	0.34	2.88
Average litter weight at birth (kg)	360	20.35	0.21	3.90	6.62	30.74
Birth weight CV (%)	360	21.24	0.31	5.86	3.64	41.96
Weaned	360	12.57	0.07	1.37	7.00	15.00
Mortality rate (%)	360	11.52	0.53	10.02	0.00	50.00
Survival rate (%)	360	88.26	0.54	10.27	50.00	100.00

Birth weight CV=Birth weight coefficient of variation, SD=Standard deviation, SE mean=Standard error mean, Min=Minimum, Max=Maximum

Parity was a significant (p < 0.05) source of variation in litter size at birth, average piglet weight, birth weight CV, number of weaned piglets, mortality, and survival rate (Table-3). Sows in parity 3–5 had greater (p < 0.05) litter size at birth (3: 17.50, 4: 17.48 and 5: 18.19) than those in lower parities (1:16.14 and 2:16.13). Birth weight in the 1^st^ (1.24 kg) and 5^th^ (1.27 kg) parity groups was significantly (p < 0.05) lower than that in parities 2–4. Birth weight in 3^rd^ parity (1.37 kg) was similar (p > 0.05) with birth weight in parities 2 (1.38 kg) and 4 (1.34 kg). The average litter weight at birth was lower (p < 0.05) in litters from parity 1 (18.53 kg) than in those from parity 2, 4, and 5 (20.95 vs. 20.74 vs. 20.03 kg). However, parity 3 had the highest (p < 0.05) average litter weight (22.07 kg). Furthermore, the average litter weight at birth was similar (p > 0.05) between sows in parity 2, 4, and 5 (20.95 vs. 20.74 vs. 20.03 kg). A similar (p > 0.05) average litter weight at birth for parity 2 (20.95 kg) and parity 3 (22.07 kg) was observed. Birth weight CV was significantly (p < 0.05) greater in higher parities (parity 4: 23.14 and 5: 25.10%) and lower in earlier parities (1: 20.77 and 2: 20.78%). There were no significant (p > 0.05) differences in the number of piglets weaned from all the parity, except for parity 5, which had the least number (p < 0.05) of weaned piglets (11.60). A significant (p < 0.05) low mortality rate was recorded for piglets whose dams were in parity 2 (8.79%); consequently, a high piglet survival rate (91.29%) was observed in the same litter. Parity 1, 3, and 4 yielded similar (p > 0.05) piglet mortality rates (11.93% vs. 13.70% vs. 12.96%). The highest piglet mortality rate (18.02%) was recorded in sows on parity 5, and a low survival rate (80.54%) was observed. There were no significant (p < 0.05) differences in survival rates between parity 1 (88.02%), 3 (86.29%), and 4 (86.95%). The total number of piglets born alive was similar (p > 0.05) among the sows of different parity.

**Table-3:** Effect of parity on pre-weaning performance of F1 Large White × Landrace piglets.

Variables	Parity				

	1^st^ (n = 96)	2^nd^ (n = 79)	3^rd^ (n = 70)	4^th^ (n = 60)	5^th^ (n = 55)
Litter size	16.14^b^ ± 0.38	16.13^b^ ± 0.46	17.50^a^ ± 0.48	17.48^a^ ± 0.49	18.19^a^ ± 0.52
Total number of born alive	15.11^a^ ± 0.37	15.15^a^ ± 0.44	16.05^a^ ± 0.46	15.45^a^ ± 0.46	15.75^a^ ± 0.49
Number of born dead	1.13^c^ ± 0.17	0.99^c^ ± 0.20	1.45^bc^ ± 0.21	2.02^ab^ ± 0.21	2.44^a^ ± 0.23
Birth weight (kg)	1.24d ± 0.01	1.38^a^ ± 0.01	1.37^ab^ ± 0.01	1.34^b^ ± 0.01	1.27^c^ ± 0.01
Average litter weight at birth (kg)	18.53^c^ ± 0.38	20.95^ab^ ± 0.42	22.07^a^ ± 0.44	20.74^b^ ± 0.48	20.03^b^ ± 0.50
Birth weight CV (%)	20.77^c^ ± 0.62	20.78^c^ ± 0.74	21.25^bc^ ± 0.78	23.14^ab^ ± 0.79	25.10^a^ ± 0.84
Number of weaned piglets	12.70^a^ ± 0.14	12.94^a^ ± 0.17	12.57^a^ ± 0.18	12.55^a^ ± 0.18	11.60^b^ ± 0.19
Mortality rate (%)	11.93^b^ ± 1.06	8.79^c^ ± 1.26	13.70^b^ ± 1.32	12.96^b^ ± 1.34	18.02^a^ ± 1.43
Survival rate (%)	88.02^b^ ± 1.07	91.29^a^ ± 1.28	86.29^b^ ± 1.34	86.95^b^ ± 1.36	80.54^c^ ± 1.45

^a,b,c^Different letters in a row indicate statistically different means at p < 0.05; Birth weight CV=Birth weight coefficient of variation

**Figure-1:**
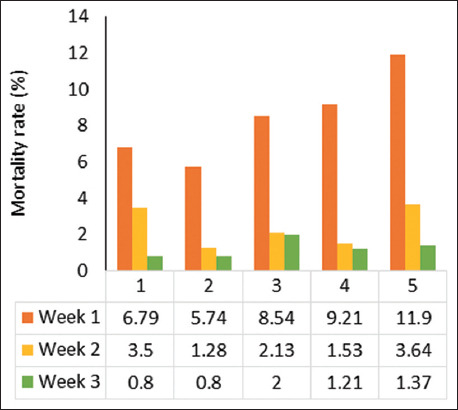
Total mortality rate at different parity over time (weeks).

The mean mortality rate of the piglets during the experimental period was 11.51%. Overall, 8.02% was recorded in the first 7 days of life, 2.41% in week 2, and 1.08% in week 3 (Figure-1). The mortality rate was significantly (p < 0.05) higher in multiparous sows in parities 3–5 (8.54%, 9.21%, and 11.90%) during week 1 compared to parity 1 (6.79%) and parity 2 (5.74%). In week 2, the mortality rate in parity 3 (2.13%) was similar (p > 0.05) to parity 2 (1.28%) and 4 (1.53%). The lowest mortality rate was observed in younger sows (parity 1 and 2), at 0.8% during week 3. The mortality rate for parity 3 (2.0%) was similar to parity 4 (1.21%) and 5 (1.37%).

The litter size at birth was highest (p < 0.05) in spring (17.72) and lowest in summer (16.47) (Table-4). Notably, litter size at birth was similar (p > 0.05) between summer (16.47), autumn (17.34), spring (17.72), and winter (16.83). However, the number of piglets born alive and the average litter weight at birth were similar (p > 0.05) in all seasons. There was no significant difference (p > 0.05) in birth weight during the autumn (1.30 kg), spring (1.29 kg), and winter (1.32 kg) seasons, whereas the highest (p < 0.05) birth weight (1.36 kg) was observed during the summer season. The birth weight CVs for summer, winter, spring, and autumn were 20.11, 21.69, 22.43, and 23.71%, respectively. Piglets born in autumn yielded higher (p < 0.05) birth weight CV (23.71%) than those born during summer (20.11%). The birth weight CV was similar (p > 0.05) in the spring, summer, and winter (20.11, 22.43, and 21.69%). The number of weaned piglets was significantly (p < 0.05) higher for those born during spring (12.82), summer (12.49), and winter (12.65) than for autumn-born piglets (11.92). The highest (p < 0.05) survival rate was observed in summer (88.40%) and winter (89.07%), and the lowest was observed in autumn (83.15%). However, the survival rate was similar (p > 0.05) for spring, summer, and winter-born piglets.

**Table-4:** Effect of season of birth on pre-weaning production performance of F1 Large White × Landrace piglets.

Variables	Spring	Summer	Autumn	Winter
Litter size	17.72^a^ ± 0.58	16.47^b^ ± 0.25	17.34^ab^ ± 0.66	16.83^ab^ ± 0.45
Total number of born alive	15.97^a^ ± 0.55	15.10^a^ ± 0.24	15.44^a^ ± 0.63	15.42^a^ ± 0.43
Number of born dead	1.75^a^ ± 0.25	1.37^a^ ± 0.11	1.90^a^ ± 0.29	1.41^a^ ± 0.20
Birth weight (kg)	1.29^b^ ± 0.01	1.36^a^ ± 0.01	1.30^b^ ± 0.02	1.32^b^ ± 0.01
Average litter weight at birth (kg)	20.75^a^ ± 0.62	20.53^a^ ± 0.27	19.32^a^ ± 0.70	20.01^a^ ± 0.46
Birth weight CV (%)	22.43^ab^ ± 0.94	20.11^b^ ± 0.41	23.71^a^ ± 1.08	21.69^ab^ ± 0.73
Weaned piglets	12.82^a^ ± 0.22	12.49^a^ ± 0.10	11.92^b^ ± 0.25	12.65^a^ ± 0.17
Mortality rate (%)	13.59^ab^ ± 1.59	11.28^b^ ± 0.69	16.59^a^ ± 1.83	10.86^b^ ± 1.23
Survival rate (%)	85.84^ab^ ± 1.61	88.40^a^ ± 0.70	83.15^b^ ± 1.85	89.07^a^ ± 1.25

^a,b^Different letters in a row indicate statistically different means at p < 0.05; Birth weight CV=Birth weight coefficient of variation

The sex of piglets significantly (p < 0.05) affected the birth weight (Table-5). Female (1.30 kg) piglets were lighter (p < 0.05) than male (1.34 kg) piglets. The variations in birth weight within litters were similar (p > 0.05) in both sexes.

**Table-5:** Effects of sex on birth weight and CV of F1 Large White×Landrace pigs.

Sex	N	Birth weight (kg)	Birth weight CV (%)
Female	2602	1.30^b^ ± 7.88	22.52^a^ ± 0.12
Male	2882	1.34^a^ ± 7.64	22.43^a^ ± 0.18

^a,b^Different letters in a row indicate statistically different means at p < 0.05; Birth weight CV: Birth weight coefficient of variation

There was a significant positive relationship between litter size and average litter weight at birth (rp = 0.576, p < 0.001), birth weight CV (rp = 0.244, p < 0.001), and mortality rate (rp = 0.378, p < 0.001) (Table-6). However, a significant negative relationship was observed between litter size and survival rate (rp = −0.384, p < 0.001) and between birth weight CV and survival rate (rp = −0.450, p < 0.001) and between mortality rate and survival rate (rp = −0.979, p < 0.001). A negative insignificant relationship was observed between average litter weight at birth and birth weight CV (rp = −0.028, p > 0.05) and survival rate (rp = −0.032, p > 0.05). However, a positive insignificant correlation was observed between average litter weight at birth and mortality rate (rp = 0.030, p > 0.05). Birth weight CV was significantly positively correlated with mortality rate (rp = 0.449, p < 0.001) and negatively correlated with survival rate (rp = −0.450, p < 0.001). A significant negative correlation was observed between mortality and survival rate (rp = −0.979, p < 0.001), which was also observed.

**Table-6:** Pearson’s moment correlation test between survival and mortality rates and independent variables (litter size, average birth weight and birth weight CV).

Traits	Litter size	Average litter weight at birth (kg)	Birth weight CV (%)	Mortality rate
Average litter weight at birth (kg)	0.576***			
Birth weight CV (%)	0.244***	−0.028^ns^		
Mortality rate (%)	0.378***	0.030^ns^	0.449***	
Survival rate (%)	−0.384***	−0.032^ns^	−0.450***	−0.979***

*** p < 0.001; NS=p > 0.05; Birth weight CV=Birth weight coefficient of variation

## Discussion

Several global studies have examined birth weight, litter uniformity, and the sizes of various pig breeds [8–10, 30]. In the study by Tucker *et al*. [31], piglet birth weights (1.24 kg) and CV (22.9%) were recorded, which correspond to the present study’s findings (1.34 kg and 21.24%). The present study’s average of 15.24 liveborn piglets and range of 5–24 exceed the reported findings from Marandu *et al*. [19], Schild *et al*. [32], and Zindove *et al*. [33]. The reports by Zindove *et al*. [33] and Marandu *et al*. [19] indicate a mean born alive of 10.13 with a range of 3–18 for purebred pigs, and 9.5 with a range of 1–19 for crossbred pigs. Klimas *et al*. [34] reported a similar weaning number difference between primiparous and multiparous sows in their study of Lithuanian White pigs. Schild *et al*. [32] documented an average of 14–18 piglets born alive among crossbred pigs. The pig breed and structure influence the live piglet count [35, 36]. The observed mean birth weight (1.34 kg) and its standard deviation (0.35) were similar to those reported by Lavary *et al*. [37] and Wientjes *et al*. [38], who reported a mean birth weight of 1.5 kg and SD of 0.4 and 1.4 kg and SD of 0.31 for the Landrace and Large White cross, respectively. The recorded birth weight CV (21.24%) and range (3.64%–41.96%) were greater than those reported by Charneca *et al*. [9] for Landrace and Large White sows (CV = 19.5%, range = 4.0–36.4). In this study, the survival rate of 88.26% was comparable to the reported 87.63% by Zindove *et al*. [39]. Similar survival rates can be achieved regardless of the number of piglets born alive. Reducing pig mortality at all stages of production has become a top priority for the pig farming industry [40]. Improving pig survival and lowering mortality at all stages of production have recently been set as priorities for the pig farming industry [40]. In comparison with other countries, the average pre-weaning mortality of the pigs in the present study (11.52%) was similar to the pig industry benchmark of Australia (11.5%) [41], and below the United States of America (14.1%) [42] and Denmark (15.2%) [43]. The present study reported a higher litter size (16.60) and total number of born alive (15.24) compared to those in Australia (litter size: 12.9, total born alive: 11.7) [41], the USA (litter size: 15.1, total born alive: 13.5) [42], but lower than Denmark (litter size: 19.8, total born alive: 17.9) [43].

The findings that the parity of the sow significantly (p < 0.05) influenced birth weight, birth weight CV, litter size, weaned piglets, mortality, and survival rate can be attributed to the fact that sows of different parities produce variable pre-parturition and post-parturition environments [33, 39]. The higher observed CV of birth weight in multiparous sows with higher parity indicates decreased litter uniformity and may result from aging-related declines in oocyte quality [44]. The quality of the oocytes determines the quality of the piglets born. This agrees with arguments put forward by Kramarenko *et al*. [45] that parity is associated with the physiological status of animals, such as growth and reproductive system development. In addition, the selection of highly prolific sows leads to more born piglets [15], consequently leading to decreased litter uniformity [17, 46]. This is due to high competition between littermates before they are born, which lowers their birth weight and decreases litter uniformity. Although sows conceive larger litters, uterine space, and blood supply have limited resources [47, 48]. The sow uterus is crowded with embryos in larger litters. As intrauterine crowding occurs, the embryos first implanted can physically restrict the development of later attaching embryos, and the embryonic competition increases with every successful embryonic attachment [49]. This may lead to decreased litter uniformity at birth.

Overall, the effect of parity on litter size may be related to the ovulation rate [17, 45]. Our results revealed that multiparous sows in their third parity produced larger litters than primiparous sows. Similar findings were observed on the increase in sow prolificacy from primiparous to second [34] and fifth to sixth parity [45]. The finding that sows in lower parity (primiparous and second parity sows) produced small litters could be a result of a lower ovulation rate in young sows than in matured sows [33]. Furthermore, the average litter weight at birth in primiparous sows was lower (18.53 kg) than in multiparous sows. These observations are in agreement with findings reported by Zindove *et al*. [50], who reported that gilt and young sows had a lighter birth weight (15.7 kg) than older sows. Multiparous sows do not require more nutrients and energy for growth and development compared to primiparous sows [51]. Therefore, their energy is directly directed to maintenance and production, which explains their higher ovulation rates than primiparous sows. During gestation, primiparous sows still need more nutrients and energy because they are still not fully mature and utilize ingested nutrients for body maintenance, maturation, and growth of the fetus [52, 53]. However, the number of born alive was not affected by parity. In other words, despite the age of the sows, hybrid sows manage to maintain a constant number of piglets born alive, although the number of born dead varies with litter size. Furthermore, the number of piglets born to death increased with litter size in these multiparous sows in their third and later stages. It has been documented that multiparous sows have more born dead piglets, which may be associated with poor muscle tone in these sows [54, 55].

A good pre-weaning survival rate was 90% or more, and a poor survival rate was 80% or less [56]. Multiparous sows in the 5^th^ parity had a lower pre-weaning survival rate for piglets in the present study. From the seventh parity onwards, Klimas *et al*. [34] noted a decline in the pre-weaning survival of piglets from sows. Our findings contrast with the minimal impact of parity on the number of weaned piglets [57]. The mortality rate among weaned piglets rises with each successive parity. An increase in the number of underweight piglets and decreased litter uniformity may be closely associated with this phenomenon [58]. Furthermore, previous studies have indicated that older sows are at risk or experience more health issues, such as (i) lameness, which causes the risk of crushing [59] and (ii) sub-functional udder morphology, making it difficult for piglets to reach teats even if the sow is lying down during suckling [60]. Inadequate colostrum intake in piglets could cause slow growth and increased pre-weaning mortality. Piglets from first-time mothers show quicker reactions when their mothers suddenly shift positions, compared to those from repeat sow mothers [61]. The study found that primiparous sows had a lower piglet mortality rate than multiparous sows. Zotti *et al*. [62] reported that the highest mortality rate was occured within the first 7 days of life. Inadequate colostrum intake and low vitality are the primary reasons for death in early life [62]. This includes starvation and sow crushing [63]. In the study, primiparous sows had fewer stillborn piglets than multiparous sows. Those studies reported similar findings as well [64]. Multiparous sows are more prone to dystocia (longer farrowing duration) than primiparous sows [65]. However, stillbirths increase with litter size [60, 66], and litter sizes >13 piglets are associated with a higher risk of stillbirth [65]. The embryonic competition increases with every successful embryonic implantation, which could hamper the development of later attaching embryos [49], leading to an increase in the number of stillbirths in large litters.

A larger litter size led to lighter piglet birth weights and less uniform litter sizes at birth. These findings are consistent with the results of earlier studies [33, 39]. Although the number of piglets born alive is an important trait for increasing the number of piglets weaned per sow per year, large litter sizes lead to a high proportion of piglets with low-birth-weights and, consequently, have a negative effect on the profitability of the industry [31]. In addition, light piglets have low-energy storage and a lower capacity to maintain their body temperature [67], taking more time to reach the udder and, therefore, struggle to select a more productive teat [68]. At birth, lightweight piglets are linked to inferior growth [39]. About 10%–15% of newborn piglets weigh under 1 kg [69]. Despite larger litters at birth, high pre-weaning mortality results in similar or smaller litter sizes at weaning [33]. Birth weight variations within litters that differ significantly are linked to decreased survival rates [70].

Multiple studies report a lower CV birth weight in primiparous sows as compared to multiparous sows [37, 45, 71, 72]. In larger litters, multiparous sows may experience lower birth weight and higher pre-weaning mortality compared to primiparous sows. Having larger litters is not beneficial when faced with significant perinatal and pre-weaning mortality risks. Reducing perinatal and pre-weaning piglet mortality requires implementing management strategies, including increasing birth weight and improving heating in farrowing housing [73]. Moving away from the sow helps the piglet conserve energy and prevent being crushed. A smaller birth weight CV can lead to higher litter sizes, better pre-weaning performance, and ultimately, increased profitability.

During farrowing season, the total live piglet count showed no significant difference (p > 0.05). The study found no significant difference (p > 0.05) in average litter weight at birth based on season of birth. Contrary to the findings of Zindove *et al*. [50], the lightest litters were born during spring, while the heaviest litters were born during autumn and winter. Litter size peaks during spring and autumn, according to the least squares plots. During hot weather, reduced feed intake in pigs results in decreased litter sizes due to diminished ovulation rates [11]. Heat stress reduces milk production and composition in piglets, leading to decreased feed intake [74]. Starvation occurs from weak suckling caused by decreased milk production in piglets [74]. Pregnant sows reduce their food intake in hot weather, resulting in weight loss, lower birth weights, and smaller litters [75]. The higher birth weight CV during autumn compared to summer increased mortality risk for smaller piglets in their autumn litters [76]. The summer season presents lower post-weaning mortality than other seasons for pigs entering growing facilities [77–79]. This is because the barn microenvironment may be difficult to manage with large daily ambient temperature variations [80]. Creating an optimal microenvironment reduces morbidity and mortality [79].

According to Riddersholm *et al*. [81], male piglets are born slightly heavier than their female counterparts. While executing a study on litter heterogeneity, the sex of the piglets has been shown to exert considerable influence [33, 82]. The piglets’ sex significantly affects fetal growth and development. Male piglets have weaker thermoregulatory capacity than female piglets [83]. The birth weight CV did not differ between male and female infants.

Significant correlations existed among litter size, birth weight CV, birth weight, and mortality rate. The proximity of smaller piglets to the sow may increase due to heightened competition, potentially resulting in devastating consequences. Tucker *et al*. [31] reported observations similar to ours. The size of litters negatively impacts their uniformity. A higher birth weight variation within a litter is linked to lower piglet survival rates due to the presence of more low-birth-weight piglets in litters with less uniformity. Compared to normal-weight piglets, low-birth-weight piglets have a greater risk of dying before weaning [84]. 24-h-old piglets with smaller body size exhibit heightened susceptibility to low viability, weakened immunity, hypothermia, and hypoglycemia [85]. Therefore, low-birth-weight piglets are at higher risk of pre-weaning mortality than their normal-weight piglet counterparts [84]. These piglets exhibit low energetic reserves and limited access to colostrum and productive teats [30, 86]. To maximize performance and profitability for commercial pig producers, a greater focus should be given to minimizing low-birth-weight piglets and exploring additional strategies for maintaining large litters.

## Conclusion

Multiparous sows yield larger litter sizes than primiparous ones. Litters from multiparous sows with higher parities exhibit decreased uniformity. With larger litters comes a decrease in uniformity among piglets at birth. A larger litter size and more variation in piglets at birth are linked to a higher pre-weaning mortality rate. To maintain productivity, producers need to strategically select females while ensuring timely culling and replacement of older sows with more efficient ones.

## Authors’ Contributions

NLB, HM, and TJM: Conceptualization and drafted and revised the manuscript. TJM, BM, and KAN: Conceptualization. TJM, BM, KAN, PAI, and MCM: Supervised and edited the manuscript. All authors have read, reviewed, and approved the final manuscript.
